# Stem Cell Recruitment of Newly Formed Host Cells via a Successful Seduction? Filling the Gap between Neurogenic Niche and Injured Brain Site

**DOI:** 10.1371/journal.pone.0074857

**Published:** 2013-09-04

**Authors:** Naoki Tajiri, Yuji Kaneko, Kazutaka Shinozuka, Hiroto Ishikawa, Ernest Yankee, Michael McGrogan, Casey Case, Cesar V. Borlongan

**Affiliations:** 1 Center of Excellence for Aging and Brain Repair, Department of Neurosurgery and Brain Repair, University of South Florida Morsani College of Medicine, Tampa, Florida, United States of America; 2 Sanbio Inc, Mountain View, California, United States of America; Stanford University School of Medicine, United States of America

## Abstract

Here, we report that a unique mechanism of action exerted by stem cells in the repair of the traumatically injured brain involves their ability to harness a biobridge between neurogenic niche and injured brain site. This biobridge, visualized immunohistochemically and laser captured, corresponded to an area between the neurogenic subventricular zone and the injured cortex. That the biobridge expressed high levels of extracellular matrix metalloproteinases characterized initially by a stream of transplanted stem cells, but subsequently contained only few to non-detectable grafts and overgrown by newly formed host cells, implicates a novel property of stem cells. The transplanted stem cells manifest themselves as pathways for trafficking the migration of host neurogenic cells, but once this biobridge is formed between the neurogenic site and the injured brain site, the grafted cells disappear and relinquish their task to the host neurogenic cells. Our findings reveal that long-distance migration of host cells from the neurogenic niche to the injured brain site can be achieved through transplanted stem cells serving as biobridges for initiation of endogenous repair mechanisms. This is the first report of a stem cell-paved “biobridge”. Indeed, to date the two major schools of discipline in stem cell repair mechanism primarily support the concept of “cell replacement” and bystander effects of “trophic factor secretion”. The present novel observations of a stem cell seducing a host cell to engage in brain repair advances basic science concepts on stem cell biology and extracellular matrix, as well as provokes translational research on propagating this stem cell-paved biobridge beyond cell replacement and trophic factor secretion for the treatment of traumatic brain injury and other neurological disorders.

## Introduction

Initially employed for in-depth examination of cell development [1], stem cells have become a cornerstone for regenerative medicine in establishing cell-based therapies for neurological disorders [2,3]. A fundamental gap in our knowledge about the mechanism underlying stem cell therapy remains unresolved. Functional recovery has been observed in experimental models of neurological disorders despite few or even absent survival of transplanted stem cells within the injured brain site [4,5]. The original concept of direct cell replacement has been challenged by the view that stem cells afford indirect rescue of the injured tissue via secretion of therapeutic molecules [6,7].

Stem cells exist even in adulthood [8], and possess the capacity to self-renew and differentiate into multiple lineages [9], contribute to normal homeostasis [10], and exert therapeutic benefits either endogenously [11–14] or following transplantation in injured organs, i.e., brain [15–21]. The subventricular zone (SVZ) of the lateral ventricles and the subgranular zone of the hippocampus dentate gyrus (DG) are the two major stem-cell niches in the adult brain [22,23], although quiescent neural stem cells (NSCs) have been detected in other brain regions [24]. Induction of stem cells after injury corresponds to a new frontier in regenerative medicine [2,3,11–21]. Indeed, laboratory studies on stem cells have recently been translated into limited clinical trials for brain disorders [25–27]. Despite these scientific advances and clinical applications, much work remains to understand the stem cell-mediated repair mechanisms in brain injury.

The present study provides evidence of a novel therapeutic feature of stem cells involving their ability to harness a biobridge between neurogenic niche and injured brain site in a traumatic brain injury (TBI) model. This biobridge expressed high levels of extracellular matrix metalloproteinases (ECM) characterized initially by a stream of transplanted stem cells, but subsequently replaced by newly formed host cells. The transplanted stem cells serve as migratory cues for host neurogenic cells, guiding their exodus from the neurogenic site towards the injured brain site. Our findings reveal that long-distance migration of host cells from the neurogenic niche to the injured brain site can be achieved through transplanted stem cells serving as biobridges for initiation of endogenous repair mechanisms.

## Materials and Methods

### Summary

This study was designed to evaluate potential therapeutic value of intracerebral transplantation of cultured Notch-induced human bone marrow-derived mesenchymal stromal cells (MSCs) (referred to as SB623, supplied by SanBio Inc.) [26,28] in an animal model of TBI. Transplantation was carried out at 7 days after TBI with functional readouts of behavioral and histological deficits conducted during the subsequent three month period after TBI. We characterized locomotor and neurological performance at baseline (prior to TBI), then at 7 days after TBI (prior to transplantation), and monthly thereafter up to three months after TBI. Following completion of behavioral testing at one month or three months after TBI, animals were euthanized by transcardial perfusion and brains harvested to histologically characterize the extent of brain damage. The stem cell engraftment and host tissue endogenous repair mechanisms (e.g., increased host cell survival in peri-TBI lesion area) were examined by immunohistochemical analyses. A total of 40 animals identified at baseline (prior to TBI surgery) as exhibiting normal behaviors (EBST: 50-60% bias swing activity; Rotorod: 60 seconds staying time on rotating rod; Bederson: at most 0-0.5 mean neurologic score), received TBI surgery as described below. Only TBI animals reaching the criterion of behavioral impairment (EBST: at least 75% bias swing activity; Rotorod: 30 seconds or less staying time on rotating rod; Bederson: at least 2.5 mean neurologic score) were randomly assigned to either SB623 transplants (n=20) or vehicle infusion (n=20) on Day 7 post-TBI. All animals were monitored monthly post-grafting for behavioral outcomes. Randomly selected animals were euthanized at one month (n=10 per group) post-TBI, and the remaining animals euthanized at three months post-TBI by transcardial perfusion with 4% paraformaldehyde. For outcome measures, transplant outcome were evaluated using the following parameters: 1) locomotor behavior via elevated body swing test (EBST) and Rotorod; (2) neurological performance via a Bederson-modified neurological examination; 3) lesion volume via hematoxylin and eosin (H&E) histologic stains; 4) graft survival via immunohistochemistry using specific antibody shown to detect human cells, and; 5) mechanism-based immunohistochemial analyses of neuroprotection and/or regeneration using antibodies directed against the grafted human cells and host cells.

### Subjects

The University of South Florida Institutional Animal Care and Use Committee approved all procedures used in this study. Animals had free access to food and water, and all were housed under normal conditions (20°C, 50% relative humidity, and a 12 hour light/dark cycle).

### TBI surgery

All surgical procedures were conducted under aseptic conditions. Adult male Sprague-Dawley (SD) rats (8-weeks old) were anesthetized with 1.5% isofluorane and checked for pain reflexes. Under deep anesthesia, animals underwent the moderate TBI model. Each animal was placed in a stereotaxic frame (anesthesia maintained via gas mask) with 1-2% isoflurane. After exposing the skull, a 4.0 mm craniectomy was performed over the left frontoparietal cortex (center at −2.0 mm anteroposterior (AP) and +2.0 mm mediolateral (ML) to bregma) [29]. A pneumatically operated metal impactor (diameter = 3.0 mm) impacted the brain at a velocity of 6.0 m/s reaching a depth of 1.0 mm below the dura mater layer and remained in the brain for 150 milliseconds. The impactor rod was angled 15° to the vertical to be perpendicular to the tangential plane of the brain curvature at the impact surface. A linear variable displacement transducer (Macrosensors, Pennsauken, NJ) connected to the impactor measured velocity and duration to verify consistency. After controlled cortical impact injury, the incision was sutured after bleeding ceased. An integrated heating pad and rectal thermometer unit with feedback control allowed maintenance of body temperature at normal limits. All animals were monitored until recovery from anesthesia. In addition, animals were weighed and observed daily for the next three consecutive days following TBI surgery, weighed twice a week thereafter, and monitored daily for health status and any signs that indicate problems or complications throughout the study.

### Grafting procedures

All surgical procedures were conducted under aseptic conditions. Animals were anesthetized with 1.5% isofluorane and checked for pain reflexes. Once deep anesthesia was achieved (by checking for pain reflexes), hair was shaved around the area of surgical incision (skull area) with enough border to prevent contaminating the operative site, followed by two surgical germicidal scrubs of site, and draping with sterile drapes. The animal was then fixed to a stereotaxic apparatus (Kopf Instruments). A 26-gauge Hamilton syringe was then lowered into a small burred skull opening (transplant coordinates were adjusted to correspond with the cortical area adjacent to the core injury site: 0.5 mm anterior and 1.0 mm lateral to bregma and 2.0 mm below the dural surface [29]). Within this single needle pass, 3 deposits of the test article (100,000 cells in 3 µl per deposit or a total of 300,000 cells in 9 µl of Plasmalyte A for 3 deposits) were made. The target area was the medial cortex which corresponded to the peri-injured cortical area, based on previously established target sites for similar stereotaxic implants. Each deposit consisted of 100,000 viable cells in 3 µl volume infused over a period of 3 minutes. Following an additional 2-minute absorption time, the needle was retracted and the wound closed stainless steel wound clip. A heating pad and a rectal thermometer allowed maintenance of body temperature at about 37°C throughout surgery and following recovery from anesthesia.

### Behavioral and neurological tests

All investigators testing the animals were blinded to the treatment condition. Animals were subjected to elevated body swing test (EBST), neurological exam, and Rotorod. EBST involved handling the animal by its tail and recording the direction of the swings. The test apparatus consisted of a clear Plexiglas box (40 x 40 x 35.5 cm). The animal was gently picked up at the base of the tail, and elevated by the tail until the animal’s nose was at a height of 2 inches (5 cm) above the surface. The direction of the swing, either left or right, was counted once the animals head moved sideways approximately 10 degrees from the midline position of the body. After a single swing, the animal was placed back in the Plexiglas box and allowed to move freely for 30 seconds prior to retesting. These steps were repeated 20 times for each animal. Intact rats display a 50% swing bias, that is, the same number of swings to the left and to the right. A 75% swing bias indicated 15 swings in one direction and 5 in the other during 20 trials. We have previously utilized the EBST, and noted that unilaterally lesioned animals display >75% biased swing activity at one month after a nigrostriatal lesion or unilateral hemispheric injury; asymmetry is stable for up to six months [3,26]. About one hour after the EBST, a modified Bederson-Neurological exam was conducted following the procedures previously described [3,26] with minor modifications. Neurologic score for each rat was obtained using 3 tests which include (1) forelimb retraction, which measured the ability of the animal to replace the forelimb after it was displaced laterally by 2 to 3 cm, graded from 0 (immediate replacement) to 3 (replacement after several seconds or no replacement); (2) beam walking ability, graded 0 for a rat that readily traversed a 2.4-cm-wide, 80-cm-long beam to 3 for a rat unable to stay on the beam for 10 seconds; and (3) bilateral forepaw grasp, which measured the ability to hold onto a 2-mm-diameter steel rod, graded 0 for a rat with normal forepaw grasping behavior to 3 for a rat unable to grasp with the forepaws. The scores from all 3 tests, which were done over a period of about 15 minutes on each assessment day, were added to give a mean neurologic deficit score (maximum possible score, 9 points divided by 3 tests = 3). After an hour of completion of the neurological exam, the animals were then subjected to the Rotorod test. The Rotorod test involved placement of the animal on an accelerating Rotorod (Accuscan, Inc.) that used a rotating treadmill that accelerates from 4 rpm to 40 rpm over a 60-second period. The total number of seconds maintained on the Rotorod was recorded and used as index of motor coordination. We have previously shown that TBI model animals exhibited significantly shorter time staying on the Rotorod compared to sham operated or normal controls. Animals were subjected to this battery of tests at baseline (prior to TBI), then at 7 days after TBI (prior to transplantation) and monthly thereafter up to three months post-TBI.

### Histology

#### Perfusion

Brain section preparation was designed to identify extent of brain damage and host cell survival. At scheduled intervals (1 month or 3 months) after TBI, randomly selected rats were euthanized (n=10 per group), and perfused by transcardial perfusion with 4% paraformaldehyde. The brains were dissected, post-fixed for overnight in 4% paraformaldehyde, then subsequently immersed in 30% sucrose. Each forebrain was cut into 40 µm thick coronal tissue sections with anterior–posterior coordinates corresponding from bregma 5.2 mm to bregma -8.8 mm per animal and subsequently processed for measurements of brain damage and analyses of cell survival in the peri-TBI lesion area (see below).

#### Measurements of brain damage

At least 4 coronal tissue sections per brain were processed for H&E staining. Every sixth coronal tissue sections per brain were collected beginning at AP -2.20 and ending at AP +0.20 anterior to the bregma, and randomly selected for measurement of cortical core and peri-injury area [29]. The indirect lesion area, in which the intact area of the ipsilateral hemisphere was subtracted from the area of the contralateral hemisphere, was calculated to reveal cerebral damage. The lesion volume was presented as a volume percentage of the lesion compared to the contralateral hemisphere.

#### Analyses of cell survival in peri-TBI lesion area

Randomly selected high powerfield corresponding to the peri-injured cortical area was used to quantitatively count host cells surviving in this region.

### Immunohistochemistry

Free floating sections were processed for immunofluorescent microscopy. Briefly, 40 µm cryostat sectioned tissues were examined at 4X magnification and digitized using a PC-based Image Tools computer program. Brain sections were blind-coded and Abercrombie’s formula was used to calculate the total number of immunopositive cells [3,26]. Cell engraftment index for SB623 was assessed using monoclonal human specific antibody (HuNu) that did not cross-react with rodent proteins. Additional brain sections were processed for mechanism-based immunohistochemical analyses of brain tissue samples focusing on cell proliferation (Ki67), migration (doublecortin or DCX) and immature neural marker (nestin).

### Zymography

A separate cohort of animals consisting of TBI plus SB623 cells, TBI plus vehicle, and control-sham operated age-matched adult SD rats (n=3 per group) was subjected to the same experimental paradigm as above, but tissues were processed for zymography, a process involving electrophoretic separation of proteins for assessment of proteolytic activity [30,31]. The tissue corresponding to the biobridge formed by the migrating cells from the SVZ to the impacted cortex was laser captured. After extraction, the tissue was placed in cryotubes and flash frozen in liquid nitrogen. The tubes were stored in a -80°C freezer until homogenization. The samples were homogenized in 450 µL of cold working buffer containing 50 mM Tris-HCl (pH 7.5), 75 mM NaCl, and 1 mM PMSF. The tissue was processed with a homogenizer for 10 minutes and centrifuged at 4°C for 20 minutes at 13000 rpm. The supernatants were separated, frozen and kept at -80°C until use. The total protein concentration was assessed by the Bradford method. On the day of the zymography, the volume equivalent to 50 µg of total protein was loaded into fresh made gelatin zymography gels. The gels were then electrophoretically separated under non-reducing conditions and 100 V. After electrophoresis the zymogram gels were washed in 125 ml 2.5% Triton twice for 20 minutes. The gels were then incubated in activation buffer (Zymogram Development Buffer, Bio-Rad, Hercules, CA) for 20 hours at 37°C. The next day, the gels were stained with Coomassie Blue R-250 Staining Solution (Bio-Rad) for 3 hours and destained for 25 minutes with Destain Solution (Bio-Rad). The gelatinolytic activity of the samples was assessed by densitometric analysis (Gel-Pro v 3.1, Media Cybernetics, Carlsbad, CA) of the bands as a relative comparison to a standard band of recombinant enzyme. To minimize inter-gel variability, all gels had a control lane loaded with 0.5 ng recombinant enzyme, which was used as a standard optical density and enzyme amount (in ng). The lytic bands identified in the zymogram gels were subjected to molecular weight identification with the use of pre-stained standard protein marker (Bio-Rad). Membranes were blocked with blotting grade blocker non-fat dry milk (Bio-Rad). After washing with 0.1% tween 20- tris-buffered saline (TTBS), the membranes were incubated with anti-matrix metalloproteinase (MMP)-9 monoclonal mouse antibody overnight at 4°C. Membranes were washed again in TTBS, incubated with secondary antibody (goat anti-mouse IgG, horseradish peroxidase conjugated antibody, Calbiochem) for one hour and finally developed with horseradish peroxidase development solution (ECL advance detection kit, Amersham). The membranes were exposed to autoradiography films (Hyblot CL, Denville Scientific Inc.). The density of the sample bands for the zymograms was expressed as maximal optical density relative to the standard band.

### Cell migration assay

Using a transwell assay, primary rat neuronal cells, PRNCs, (embryos at Day 18; BrainBits) (1×10^5^ cells/well) seeded onto the upper chamber of a Boyden chamber (Costar Transwell assay, Corning, NY, USA) supplemented with NbActive4 (BrainBits) in the absence of antibiotics. The chamber was placed in a 24-well plate containing confluent SB623 cells (1×10^5^ cells/well) and starved with serum-free DMEM/F-12 medium in the presence or absence of Cyclosporine-A (a known MMP-9 inhibitor; 10^4^ ng/mL in dimethyl sulfoxide; Sigma-Aldrich Inc., St Louis, MO, USA) for 24 h in the cell incubator. Next, the upper chamber was removed and wiped clean, then the lower side of the filter was washed and fixed in 4% paraformaldehyde. For quantification, migratory cells that reached the lower chamber and attached to the lower side of the filter were counted from five randomly captured microscopic fields (X400) and averaged for each treatment condition. This migratory assay was performed in triplicates.

## Results

**Figure 1:**
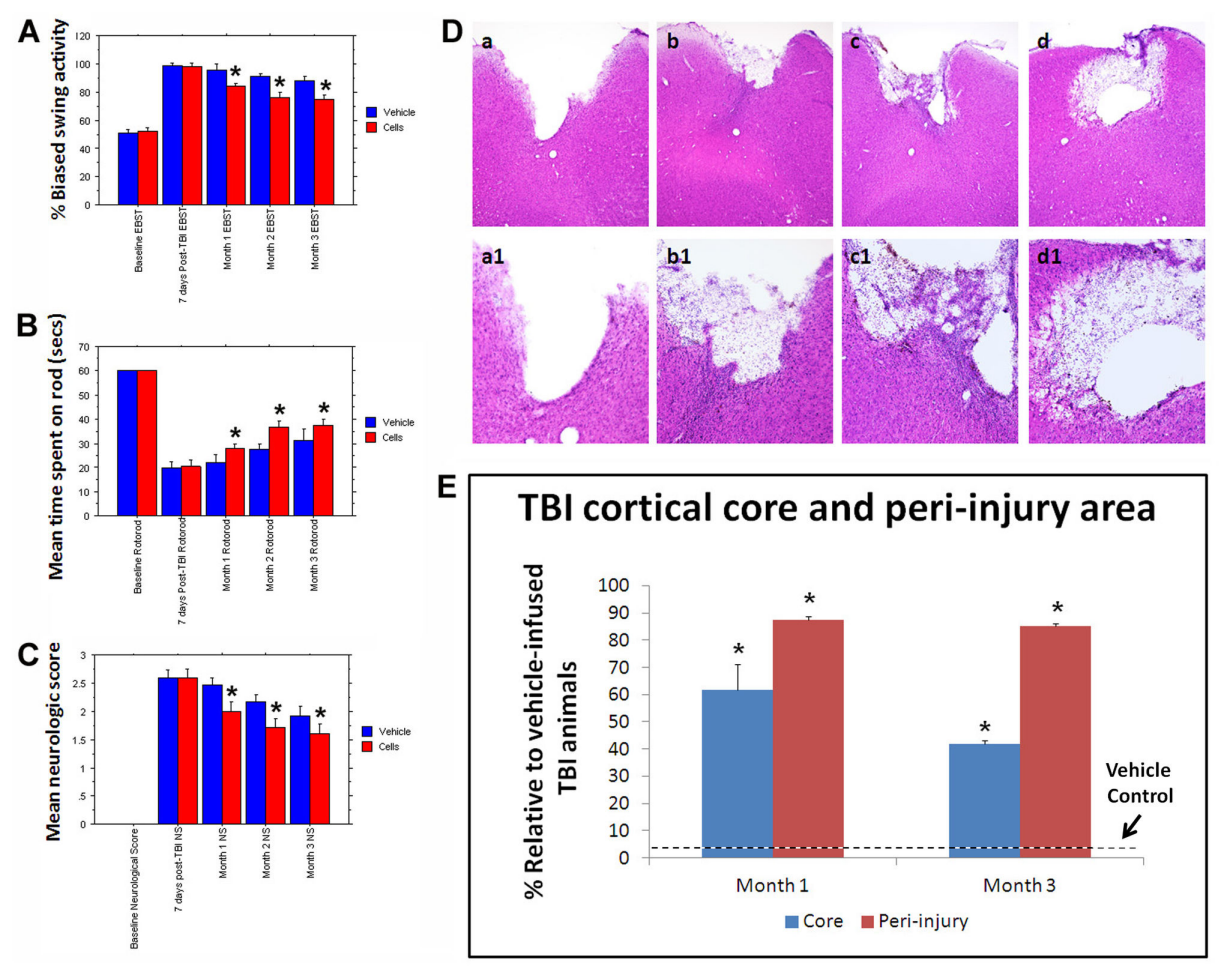
Behavioral tests (performed by two investigators blinded to the treatment condition throughout the study) were initially conducted at baseline (i.e., prior to brain insult) and revealed that all adult SD rats included in this study displayed normal behaviors (A, B, and C). At 7 days after TBI, the same behavioral tests showed that TBI produced significant impairments in motor and neurological tasks. At one month, two months, and three months post-TBI, transplanted animals displayed significantly improved motor and neurological functions compared to traumatically injured animals that received vehicle only. These behavioral improvements were accompanied by reduction in TBI core and peri-injury cell death (D and E) as revealed by H&E staining (a and b correspond to vehicle and transplant respectively at one month post-treatment, while c and d represent vehicle and transplant respectively at three months post-treatment. a-d are at 10X while a’-d’ at 20X magnification). Asterisks (*) indicate significant improvements in behavioral and histological deficits in TBI transplanted cells compared to TBI animals that received vehicle only (p’s < 0.05).

**Figure 2:**
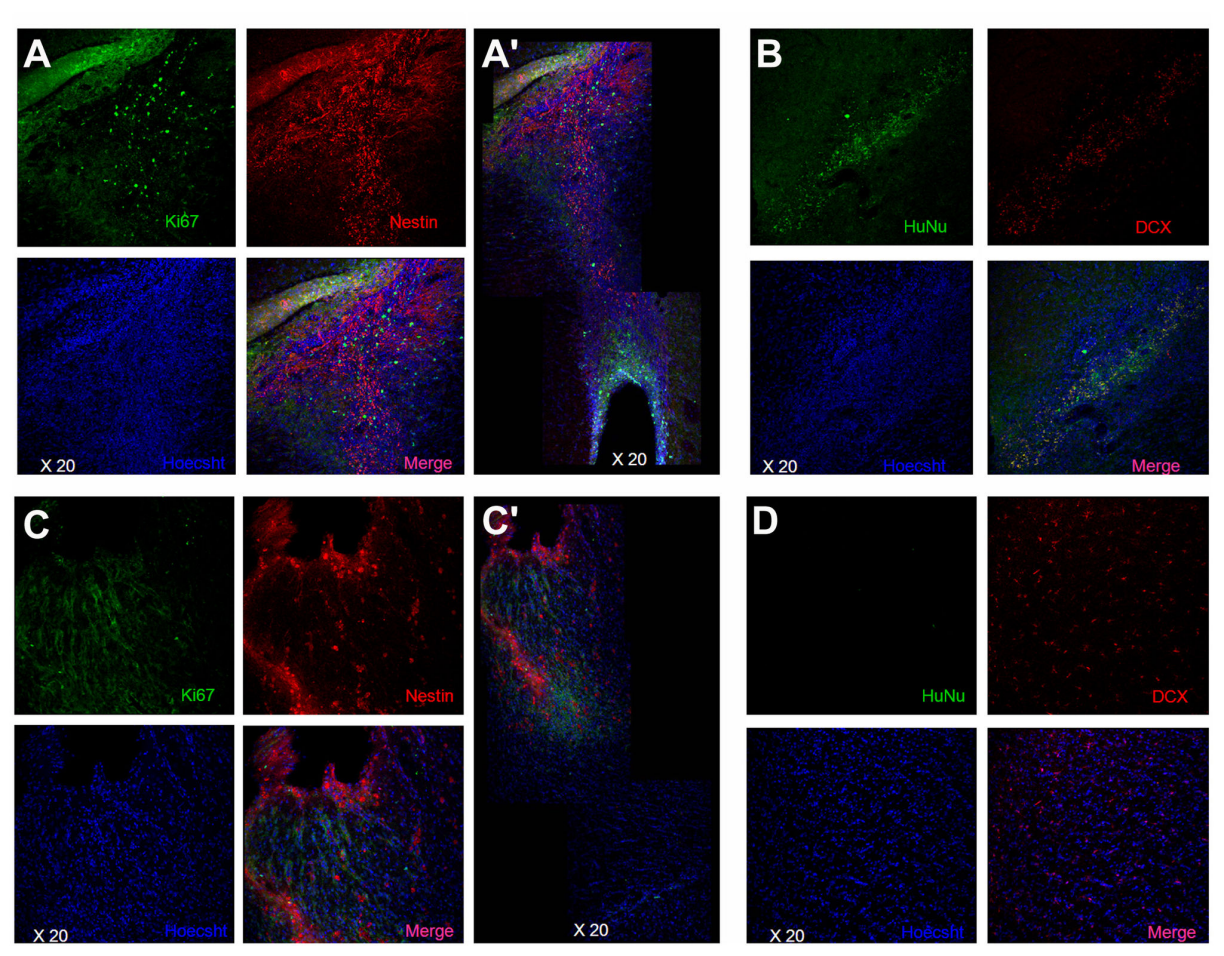
The biobridge between SVZ and impacted cortex consists of highly proliferative, neurally committed, and migratory cells. At one month post-TBI, confocal microscopy revealed a surge of proliferative Ki67 positive cells and immature neurally nestin labeled cells in the peri-injured cortical areas (A) and subventricular zone (A’), with a stream of migrating cells (DCX) along the corpus callosum (B) in TBI animals that received the stem cell transplants. In contrast those that received vehicle alone displayed limited cell proliferation (C), neural differentiation (C’), and scattered migration in the peri-injured cortical areas (D) and almost absent expression of newly formed cells in the SVZ (C’).

**Figure 3:**
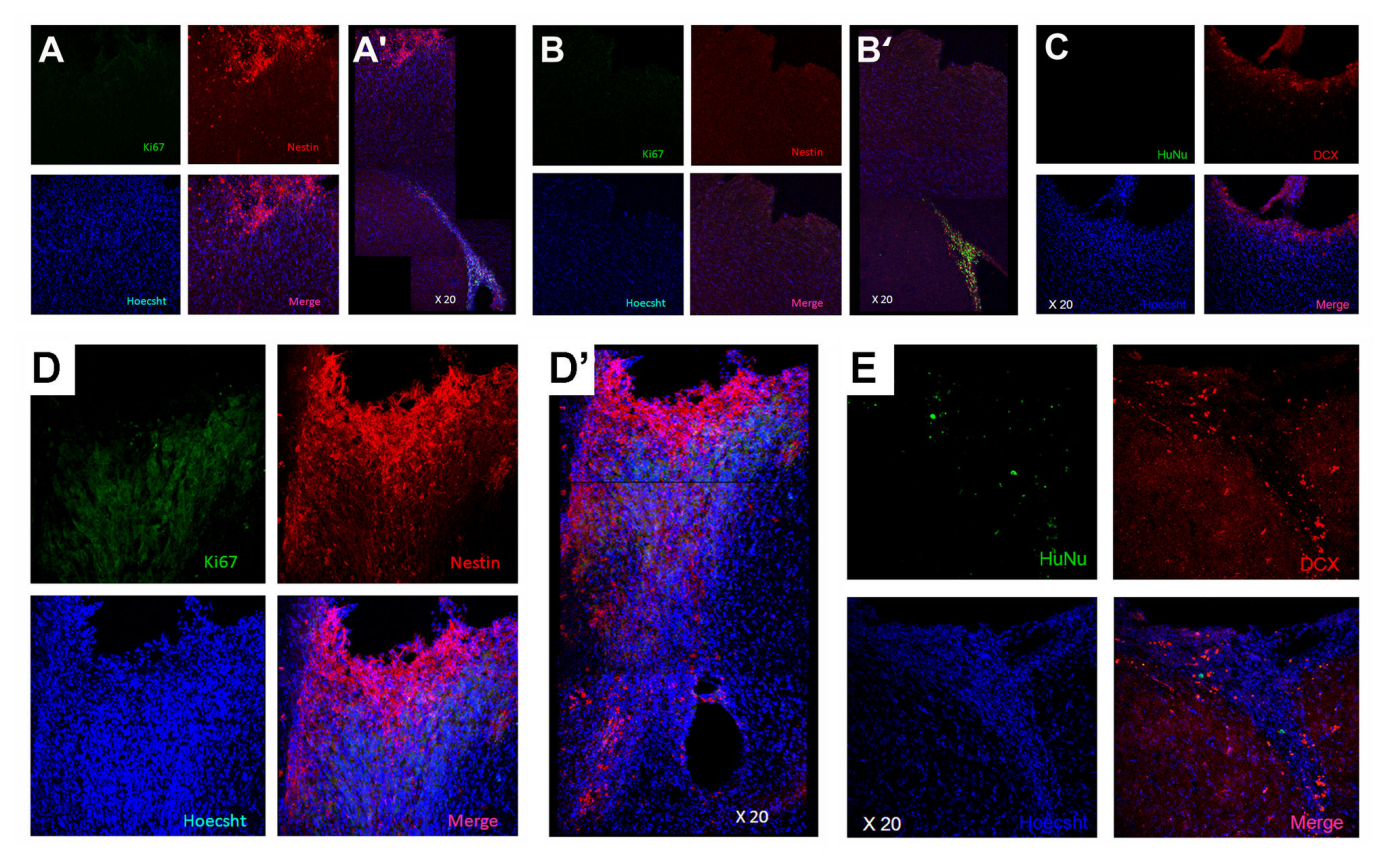
At three months post-TBI, the brains from vehicle-infused animals displayed a disparate pattern of cell fate in that the newly formed Ki67 positive and nestin labeled cells were sequestered within the corpus callosum (A) and the SVZ (B) and only sporadic cells were able to reach the impacted cortex (A’ and B’), with likely resident DCX cells seen around the impacted cortex (C). In contrast, at three months post-TBI, the brains from transplanted animals exhibited a much more massive cell proliferation and neural differentiation encasing the peri-injured cortical areas accompanied by a solid stream of nestin (D, D’) and DCX labeled cells (E) migrating not just along, but across the corpus callosum from the SVZ to the impacted cortex.

**Figure 4:**
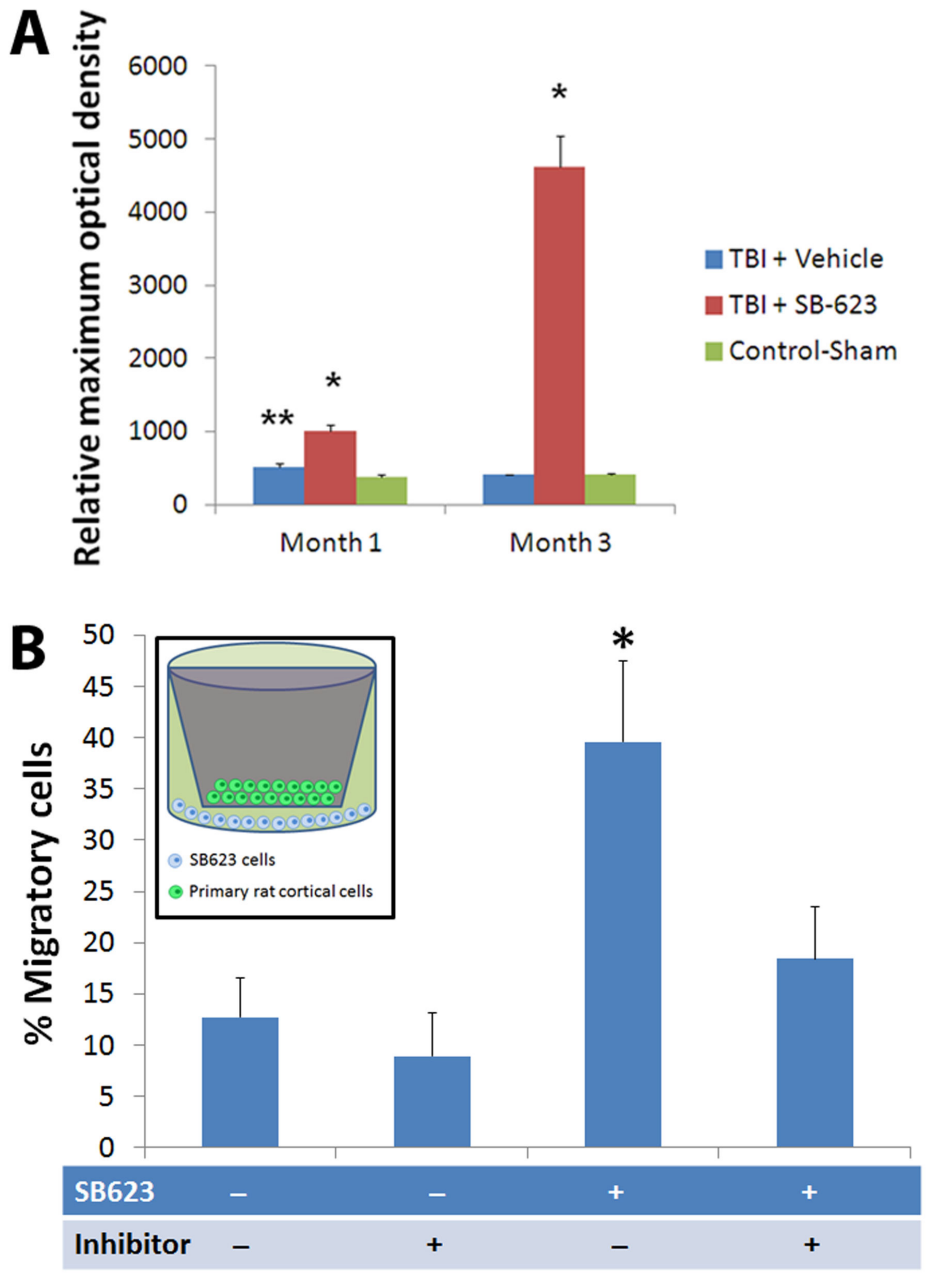
Laser-captured biobridge, corresponding to the brain tissue between SVZ and impacted cortex, expressed high levels of MMP-9 gelatinolytic activities at one month and three months post-TBI in animals transplanted with SB623 which were significantly higher than those TBI animals that received vehicle only or sham-operated animals (*p’s< 0.05 vs. vehicle or sham; Panel A). Although vehicle-infused TBI animals also showed a significantly upregulated MMP-9 gelatinolytic activity at one month post-TBI (**p< 0.05 vs. sham), the level of this neurovascular proteinase activity reverted back to control-sham levels at three months post-TBI. Each bar represents the mean ± standard deviation from n=3 per treatment group for each time point. Next, to further reveal that SB623 cells promoted cell migration via an ECM-mediated mechanism, primary rat cortical cells were either grown alone or co-cultured with SB623 in the presence or absence of the MMP-9 inhibitor Cyclosporine-A (Panel **B**). Migratory cell assay (see inset) revealed significantly enhanced migration of primary rat cortical cells into the chamber that contained SB623, which was significantly suppressed by treatment with the inhibitor (*p< 0.05 vs. all other treatment conditions). The absence of SB623 and inhibitor in the cell culture condition, the treatment of the inhibitor alone, and the combined treatment of SB623 and inhibitor did not significantly differ in the resulting cell migratory potential.

Adult male SD rats were initially evaluated in motor and neurological tests (all performed by two investigators blinded to the treatment condition throughout the study) to confirm that all animals included here were displaying normal behaviors at baseline (i.e., prior to brain insult). Animals were exposed to experimental TBI, then seven days later subjected to the same behavioral tests to confirm the typical TBI-induced motor and neurological impairments, and thereafter (also at 7 days post-TBI) assigned in a random fashion to receive either stereotaxic transplants of either SB623 cells [26,32] or vehicle infusion into the cortex (see Methods). At one month and three months post-TBI, transplanted animals displayed significantly improved motor and neurological functions coupled with significantly reduced damage to the cortical core and peri-injured cortical areas compared to traumatically injured animals that received vehicle only (Figure 1). These behavioral and histological improvements were achieved with modest graft survival of 0.60% and 0.16% at one month and three months post-TBI, respectively. Based on the robust functional recovery despite lack of graft persistence, we next examined the status of the host tissue. At one month post-TBI, immunofluorescent and confocal microscopy revealed a surge of endogenous cell proliferation (Ki67) and immature neural differentiation (nestin) in the peri-injured cortical areas and SVZ, with a stream of migrating cells (DCX) along the corpus callosum (CC) of the transplanted animals, while those that received vehicle alone displayed limited cell proliferation, neural differentiation, and scattered migration in the peri-injured cortical areas and almost absent expression of newly formed cells in the SVZ (Figure 2). At three months post-TBI, the brains from transplanted animals exhibited a much more massive cell proliferation and neural differentiation encasing the peri-injured cortical areas (CTX) accompanied by a solid stream of neuronally labeled cells (nestin as well as DCX) migrating not just along but across the CC from the SVZ to the impacted CTX (Figure 3). In contrast, the brains from vehicle-infused animals while producing a much more elevated cell proliferation, showed that newly formed cells were “trapped” within the SVZ and the CC and only sporadic cells were able to reach the impacted CTX (Figure 3). Quantitative analyses of Ki67, nestin and DCX immunolabeled cells in SVZ, DG, CC and CTX revealed statistically significant differences between transplanted and vehicle-infused animals (Figure S1). We next focused on the biobridge formed by the migrating cells from the SVZ to the impacted cortex and laser captured this corresponding tissue in a separate cohort of animals consisting of TBI plus SB623 cells, TBI plus vehicle, and control-sham operated age-matched adult SD rats (n=3 per group) using the same experimental paradigm as above. Zymographic assays revealed two-fold and nine-fold upregulation of the MMP-9 expression/activity in TBI transplanted animals at one month and three months post-transplantation compared to vehicle-infused TBI animals or control-sham operated animals (Figure 4A). Parallel *in vitro* studies provided further evidence that SB623 cells promoted cell migration via an ECM-mediated mechanism (Figure 4B).

## Discussion

**Figure 5:**
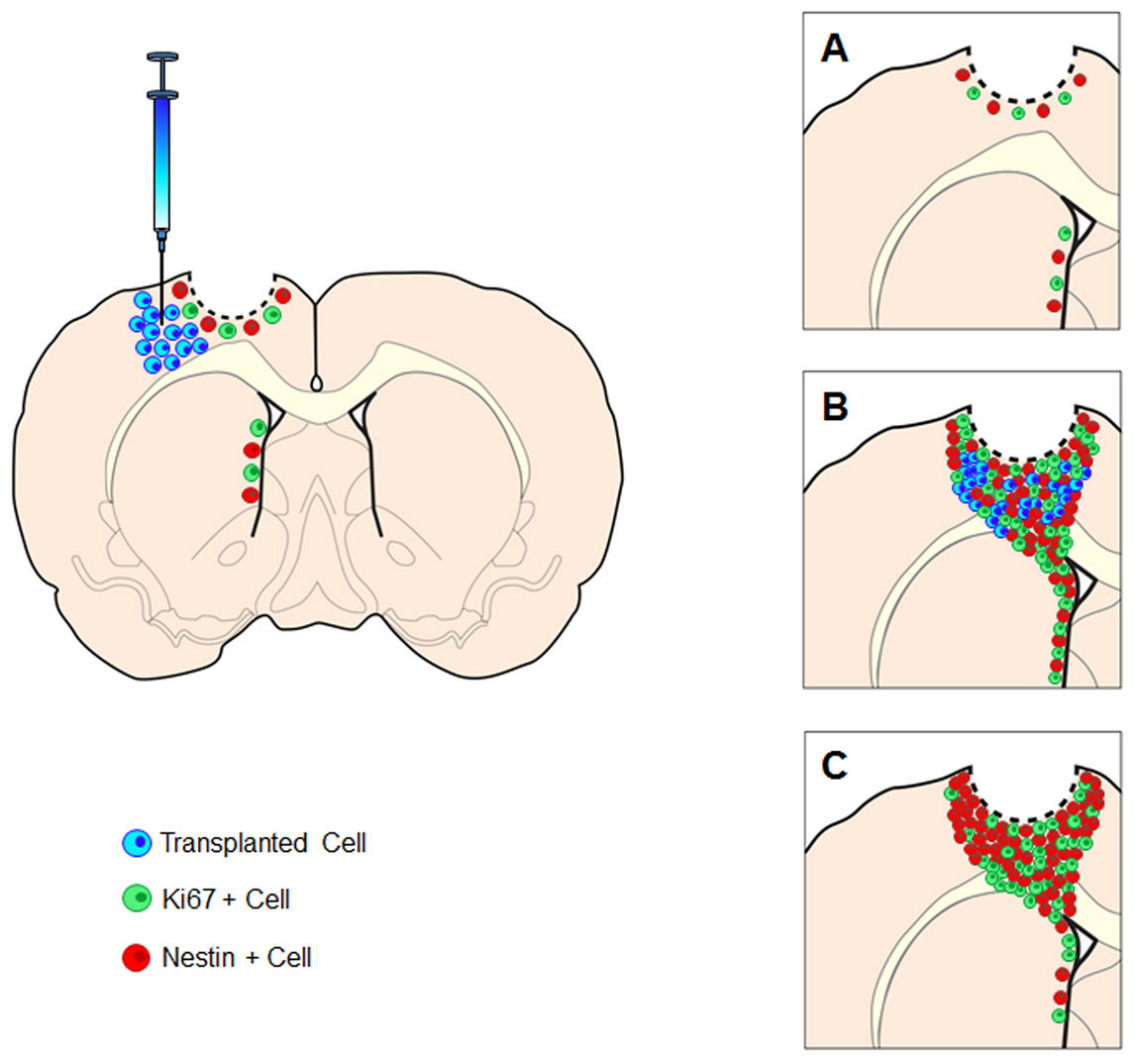
After TBI, endogenous repair mechanisms commenced, but are limited to the neurogenic SVZ and to a few quiescent resident neurogenic cells around the impacted cortex (A). This endogenous repair process is not sufficient to mount a robust and stable defense against the TBI-induced cell death cascade unless exogenous stem cells are introduced. A physical gap between the neurogenic SVZ and the non-neurogenic, impacted cortex prevents migration of neurogenic cells to the injured cortex. Transplantation of stem cells into the peri-injured cortical areas creates a neurovascular matrix of biobridge to bootleg newly formed endogenous cells from the SVZ to the peri-injured cortex (B). Once the biobridge is established, the endogenous repair mechanism is maintained by newly formed host cells even in the absence of stem cells (C). Such transplant-paved biobridge between neurogenic and non-neurogenic sites allows endogenous neurogenic cells to reach injury-specific brain sites.

The present results revealed that SB623 transplants remodeled the traumatically injured brain by harnessing a biobridgebetween SVZ and the peri-injured cortex (Figure 5). This new mechanism of stem cell therapy opens the possibility of creating similar biobridges between neurogenic and non-neurogenic sites to facilitate injury-specific migration of cells across tissues that otherwise are non-conducive barriers against cell motility.

A Phase I/IIa transplantation study of SB623 cells in chronic stroke patients is underway. The clinical product entails allogeneic SB623 cells. In cell culture and animal models of brain disorders, SB623 cells have been shown to attenuate behavioral and histological deficits associated with stroke, spinal cord injury, and Parkinson’s disease [33–35]. The present study is designed to extend the utility of SB623 in TBI. The US FDA recently approved a limited clinical trial of transplanting SB623 cells in TBI, in part based on the data being reported here. Thus, a major human implication impetus in this study was to provide the preclinical basis for initiating a clinical trial of SB623 in TBI.

The novel finding of SB623-facilitated migration of endogenous cells via a biobridge implicates the active role of MMPs and ECMs in stroke pathology [36,37] and their increasingly recognized role as therapeutic targets for stroke [38,39]. A variety of stem cells, including those derived from umbilical cord blood, peripheral blood, and adult brain, have been demonstrated to alter levels and functions of MMPs and ECMs [40–42], which would suggest their potential to similarly serve as biobridges as seen with the present Notch-induced SB623 MSCs.

Although neurogenic niches in the adult brain, such as the SVZ, have now been documented to exist and demonstrated to be critical in the repair of the stroke brain [43–47], a key limiting factor for endogenous repair is the successful migration of these newly formed host cells to reach the ischemic brain area. Our present results suggest that SB623 cell transplantation boosted endogenous repair mechanisms by guiding the migration of new cells from the neurogenic SVZ, across a non-neurogenic brain area, and eventually reaching the site of injury. The fundamental mechanism of action of SB623 cells involves their capacity to form biobridges consisting of MMPs and ECMs which serve as a gateway to ferry the newly formed cells from the neurogenic niche into the ischemic tissue. Although the grafted SB623 pioneered the formation of these biobridges, they subsequently relinquished these biobridges to the endogenous stem cells, altogether facilitating the host brain remodeling process. Our findings directly advance the concept of a biobridge mechanism as a robust stem cell-mediated brain repair strategy in TBI, and provide pivotal guidance on the translational applications of cell therapy in TBI patients. Future studies require closely monitoring the long-term efficacy and safety of SB623 cell therapy in chronic TBI animals in order to further optimize the conduct of the clinical trial of these cells in TBI patients.

A basic knowledge gap in functional restoration after stem cell transplantation is the elusive demonstration of integration of grafted cells into the recipient brain tissue and their subsequent interaction with host cells. The cellular interaction between the transplanted cell and host cell becomes extremely essential when graft survival is mediocre, indicating that for robust and stable therapeutic benefits an endogenous repair mechanism must be set in motion by the graft, in particular finding a way for the host cells to reach their destination even across non-neurogenic and injured tissues. MMPs have been implicated in recovery in chronic brain injury [32], with MMP inhibition abrogating neurogenic migration from SVZ into damaged tissues and retarding neurovascular remodeling [48]. Stem cells may serve as biobridges expressing MMP profiles that recapitulate the neurovascular unit abetting the transplant-mediated host cell migration towards injured brain areas in affording functional recovery in TBI.

## Supporting Information

Figure S1 **Quantifications of Ki67, nestin and DCX labeled cells are shown in panels A, B, and C, respectively.** Asterisks (*) indicate significant increase in the number of phenotypically labeled cells counted per high-power field view (28,800 µm^2^) selected at random in the region of interest in TBI animals transplanted with SB623 cells compared to TBI animals that received vehicle only (p’s < 0.05).
(TIF)

## References

[1] Joyner AL, Skarnes WC, Rossant J (1989) Production of a mutation in mouse En-2 gene by homologous recombination in embryonic stem cells. Nature 338: 153-156. doi:10.1038/338153a0. PubMed: 2563902. 2563902 10.1038/338153a0

[2] Yasuhara T, Matsukawa N, Hara K, Yu G, Xu L et al. (2006) Transplantation of human neural stem cells exerts neuroprotection in a rat model of Parkinson’s disease. J Neurosci 26: 12497-12511. doi:10.1523/JNEUROSCI.3719-06.2006. PubMed: 17135412. 17135412 10.1523/JNEUROSCI.3719-06.2006 PMC6674904

[3] Yasuhara T, Hara K, Maki M, Mays RW, Deans RJ et al. (2008) Intravenous grafts recapitulate the neurorestoration afforded by intracerebrally delivered multipotent adult progenitor cells in neonatal hypoxic-ischemic rats. J Cereb Blood Flow Metab 28: 1804-1810. doi:10.1038/jcbfm.2008.68. PubMed: 18594556. 18594556 10.1038/jcbfm.2008.68 PMC2587070

[4] Borlongan CV, Hadman M, Sanberg CD, Sanberg PR (2004) Central nervous system entry of peripherally injected umbilical cord blood cells is not required for neuroprotection in stroke. Stroke 35: 2385-2389. doi:10.1161/01.STR.0000141680.49960.d7. PubMed: 15345799. 15345799 10.1161/01.STR.0000141680.49960.d7

[5] Pastori C, Librizzi L, Breschi GL, Regondi C, Frassoni C et al. (2008) Arterially perfused neurosphere-derived cells distribute outside the ischemic core in a model of transient focal ischemia and reperfusion in vitro. PLOS ONE 3: e2754. doi:10.1371/journal.pone.0002754. PubMed: 18648648. 18648648 10.1371/journal.pone.0002754 PMC2453234

[6] Redmond DE Jr, Bjugstad KB, Teng YD, Ourednik V, Ourednik J et al. (2007) Behavioral improvement in a primate Parkinson’s model is associated with multiple homeostatic effects of human neural stem cells. Proc Natl Acad Sci U S A 104: 12175-12180. doi:10.1073/pnas.0704091104. PubMed: 17586681. 17586681 10.1073/pnas.0704091104 PMC1896134

[7] Lee JP, Jeyakumar M, Gonzalez R, Takahashi H, Lee PJ et al. (2007) Stem cells act through multiple mechanisms to benefit mice with neurodegenerative metabolic disease. Nat Med 13: 439-447. doi:10.1038/nm1548. PubMed: 17351625. 17351625 10.1038/nm1548

[8] Ma DK, Marchetto MC, Guo JU, Ming GL, Gage FH et al. (2010) Epigenetic choreographers of neurogenesis in the adult mammalian brain. Nat Neurosci 13: 1338-1344. doi:10.1038/nn.2672. PubMed: 20975758. 20975758 10.1038/nn.2672 PMC3324277

[9] Hong SH, Rampalli S, Lee JB, McNicol J, Collins T et al. (2011) Cell fate potential of human pluripotent stem cells is encoded by histone modifications. Cell Stem Cell 9: 24-36. doi:10.1016/j.stem.2011.06.002. PubMed: 21726831. 21726831 10.1016/j.stem.2011.06.002

[10] Kim Y, Sharov AA, McDole K, Cheng M, Hao H et al. (2011) Mouse B-type lamins are required for proper organogenesis but not by embryonic stem cells. Science 334: 1706-1710. 22116031 10.1126/science.1211222 PMC3306219

[11] Borlongan CV (2011) Bone marrow stem cell mobilization in stroke: a ‘bonehead’ may be good after all! Leukemia 25: 1674-1686. doi:10.1038/leu.2011.167. PubMed: 21727900. 21727900 10.1038/leu.2011.167 PMC3291108

[12] Barha CK, Ishrat T, Epp JR, Galea LA, Stein DG (2011) Progesterone treatment normalizes the levels of cell proliferation and cell death in the dentate gyrus of the hippocampus after traumatic brain injury. Exp Neurol 231: 72-81. doi:10.1016/j.expneurol.2011.05.016. PubMed: 21684276. 21684276 10.1016/j.expneurol.2011.05.016 PMC3153556

[13] Jaskelioff M, Muller FL, Paik JH, Thomas E, Jiang S et al. (2011) Telomerase reactivation reverses tissue degeneration in aged telomerase-deficient mice. Nature 469: 102-106. doi:10.1038/nature09603. PubMed: 21113150. 21113150 10.1038/nature09603 PMC3057569

[14] Wang L, Chopp M, Teng H, Bolz M, Francisco MA et al. (2011) Tumor necrosis factor α primes cerebral endothelial cells for erythropoietin-induced angiogenesis. J Cereb Blood Flow Metab 31: 640-647. doi:10.1038/jcbfm.2010.138. PubMed: 20700128. 20700128 10.1038/jcbfm.2010.138 PMC3049518

[15] Andres RH, Horie N, Slikker W, Keren-Gill H, Zhan K et al. (2011) Human neural stem cells enhance structural plasticity and axonal transport in the ischaemic brain. Brain 134: 1777-1789. doi:10.1093/brain/awr094. PubMed: 21616972. 21616972 10.1093/brain/awr094 PMC3102243

[16] Liu Z, Li Y, Zhang RL, Cui Y, Chopp M (2011) Bone marrow stromal cells promote skilled motor recovery and enhance contralesional axonal connections after ischemic stroke in adult mice. Stroke 42: 740-744. doi:10.1161/STROKEAHA.110.607226. PubMed: 21307396. 21307396 10.1161/STROKEAHA.110.607226 PMC3060040

[17] Mazzocchi-Jones D, Döbrössy M, Dunnett SB (2009) Embryonic striatal grafts restore bi-directional synaptic plasticity in a rodent model of Huntington's disease. Eur J Neurosci 30: 2134-2142. doi:10.1111/j.1460-9568.2009.07006.x. PubMed: 20128850. 20128850 10.1111/j.1460-9568.2009.07006.x

[18] Lee HS, Bae EJ, Yi SH, Shim JW, Jo AY et al. (2010) Foxa2 and Nurr1 synergistically yield A9 nigral dopamine neurons exhibiting improved differentiation, function, and cell survival. Stem Cells 28: 501-512. PubMed: 20049900. 20049900 10.1002/stem.294

[19] Hargus G, Cooper O, Deleidi M, Levy A, Lee K et al. (2010) Differentiated Parkinson patient-derived induced pluripotent stem cells grow in the adult rodent brain and reduce motor asymmetry in Parkinsonian rats. Proc Natl Acad Sci U S A 107: 15921-15926. PubMed: 20798034. 20798034 10.1073/pnas.1010209107 PMC2936617

[20] Yasuda A, Tsuji O, Shibata S, Nori S, Takano M et al. (2011) Significance of remyelination by neural stem/progenitor cells transplanted into the injured spinal cord. Stem Cells 29: 1983-1994. doi:10.1002/stem.767. PubMed: 22028197. 22028197 10.1002/stem.767

[21] Mezey E (2011) The therapeutic potential of bone marrow-derived stem cells. J Cell Biochem 112: 2683-2687. doi:10.1002/jcb.23216. PubMed: 21678464. 21678464 10.1002/jcb.23216 PMC4601607

[22] Sanai N, Nguyen T, Ihrie RA, Mirzadeh Z, Tsai HH et al. (2011) Corridors of migrating neurons in the human brain and their decline during infancy. Nature 478: 382-386. doi:10.1038/nature10487. PubMed: 21964341. 21964341 10.1038/nature10487 PMC3197903

[23] Carlén M, Meletis K, Göritz C, Darsalia V, Evergren E et al. (2009) Forebrain ependymal cells are Notch-dependent and generate neuroblasts and astrocytes after stroke. Nat Neurosci 12: 259-267. doi:10.1038/nn.2268. PubMed: 19234458. 19234458 10.1038/nn.2268

[24] Robel S, Berninger B, Götz M (2011) The stem cell potential of glia: lessons from reactive gliosis. Nat Rev Neurosci 12: 88-104. doi:10.1038/nrn2978. PubMed: 21248788. 21248788 10.1038/nrn2978

[25] Seol HJ, Jin J, Seong DH, Joo KM, Kang W et al. (2011) Genetically engineered human neural stem cells with rabbit carboxyl esterase can target brain metastasis from breast cancer. Cancer Lett 311: 152-159. doi:10.1016/j.canlet.2011.07.001. PubMed: 21868150. 21868150 10.1016/j.canlet.2011.07.001

[26] Yasuhara T, Matsukawa N, Hara K, Maki M, Ali MM et al. (2009) Notch-induced rat and human bone marrow stromal cell grafts reduce ischemic cell loss and ameliorate behavioral deficits in chronic stroke animals. Stem Cells Dev 18: 1501-1514. doi:10.1089/scd.2009.0011. PubMed: 19301956. 19301956 10.1089/scd.2009.0011

[27] Pollock K, Stroemer P, Patel S, Stevanato L, Hope A et al. (2006) A conditionally immortal clonal stem cell line form human cortical neuroepithelium for the treatment of ischemic stroke. Exp Neurol 199: 143-155. doi:10.1016/j.expneurol.2005.12.011. PubMed: 16464451. 16464451 10.1016/j.expneurol.2005.12.011

[28] Dezawa M, Kanno H, Hoshino M, Cho H, Matsumoto N et al. (2004) Specific induction of neuronal cells from bone marrow stromal cells and application for autologous transplantation. J Clin Invest 113: 1701-1710. doi:10.1172/JCI20935. PubMed: 15199405. 15199405 10.1172/JCI20935 PMC420509

[29] Paxinos G, Watson C (2007) The Rat Brain in Stereotaxic Coordinates: Hard Cover Edition. Academic Press.

[30] Hawkes SP, Li H, Taniguchi GT (2010) Zymography and reverse zymography for detecting MMPs and TIMPs. Methods Mol Biol 622: 257-269. doi:10.1007/978-1-60327-299-5_16. PubMed: 20135288. 20135288 10.1007/978-1-60327-299-5_16

[31] Machado LS, Kozak A, Ergul A, Hess DC, Borlongan CV et al. (2006) Delayed minocycline inhibits ischemia-activated matrix metalloproteinases 2 and 9 after experimental stroke. BMC Neurosci 7: 56. doi:10.1186/1471-2202-7-56. PubMed: 16846501. 16846501 10.1186/1471-2202-7-56 PMC1543649

[32] Zhao BQ, Tejima E, Lo EH (2007) Neurovascular proteases in brain injury, hemorrhage and remodeling after stroke. Stroke 38: 748-752. doi:10.1161/01.STR.0000253500.32979.d1. PubMed: 17261731. 17261731 10.1161/01.STR.0000253500.32979.d1

[33] Aizman I, Tate CC, McGrogan M, Case CC (2009) Extracellular matrix produced by bone marrow stromal cells and by their derivative, SB623 cells, supports neural cell growth. J Neurosci Res 87: 3198-3206. doi:10.1002/jnr.22146. PubMed: 19530164. 19530164 10.1002/jnr.22146

[34] Tate CC, Fonck C, McGrogan M, Case CC (2010) Human mesenchymal stromal cells and their derivative, SB623 cells, rescue neural cells via trophic support following in vitro ischemia. Cell Transplant 19: 973-984. doi:10.3727/096368910X494885. PubMed: 20350349. 20350349 10.3727/096368910X494885

[35] Dao M, Tate CC, McGrogan M, Case CC (2013) Comparing the angiogenic potency of naive marrow stromal cells and Notch-transfected marrow stromal cells. J Transl Med 11: 81. doi:10.1186/1479-5876-11-81. PubMed: 23531336. 23531336 10.1186/1479-5876-11-81 PMC3615967

[36] Park KP, Rosell A, Foerch C, Xing C, Kim WJ et al. (2009) Plasma and brain matrix metalloproteinase-9 after acute focal cerebral ischemia in rats. Stroke 40: 2836-2842. doi:10.1161/STROKEAHA.109.554824. PubMed: 19556529. 19556529 10.1161/STROKEAHA.109.554824 PMC3712850

[37] del Zoppo GJ, Frankowski H, Gu YH, Osada T, Kanazawa M et al. (2012) Microglial cell activation is a source of metalloproteinase generation during hemorrhagic transformation. J Cereb Blood Flow Metab 32: 919-932. doi:10.1038/jcbfm.2012.11. PubMed: 22354151. 22354151 10.1038/jcbfm.2012.11 PMC3345906

[38] Tejima E, Guo S, Murata Y, Arai K, Lok J et al. (2009) Neuroprotective effects of overexpressing tissue inhibitor of metalloproteinase TIMP-1. J Neurotrauma 26: 1935-1941. doi:10.1089/neu.2009.0959. PubMed: 19469687. 19469687 10.1089/neu.2009.0959 PMC2822804

[39] Chen J, Cui X, Zacharek A, Cui Y, Roberts C et al. (2011) White matter damage and the effect of matrix metalloproteinases in type 2 diabetic mice after stroke. Stroke 42: 445-452. doi:10.1161/STROKEAHA.110.596486. PubMed: 21193743. 21193743 10.1161/STROKEAHA.110.596486 PMC3108495

[40] Barkho BZ, Munoz AE, Li X, Li L, Cunningham LA et al. (2008) Endogenous matrix metalloproteinase (MMP)-3 and MMP-9 promote the differentiation and migration of adult neural progenitor cells in response to chemokines. Stem Cells 26: 3139-3149. doi:10.1634/stemcells.2008-0519. PubMed: 18818437. 18818437 10.1634/stemcells.2008-0519 PMC2758553

[41] Sobrino T, Pérez-Mato M, Brea D, Rodríguez-Yáñez M, Blanco M et al. (2012) Temporal profile of molecular signatures associated with circulating endothelial progenitor cells in human ischemic stroke. J Neurosci Res 90: 1788-1793. doi:10.1002/jnr.23068. PubMed: 22513751. 22513751 10.1002/jnr.23068

[42] Lin CH, Lee HT, Lee SD, Lee W, Cho CW et al. (2013) Role of HIF-1alpha-activated Epac1 on HSC-mediated neuroplasticity in stroke model. Neurobiol Dis 58C: 76-91. 10.1016/j.nbd.2013.05.006 23702312

[43] Ekdahl CT, Kokaia Z, Lindvall O (2009) Brain inflammation and adult neurogenesis: the dual role of microglia. Neuroscience 158: 1021-1029. doi:10.1016/j.neuroscience.2008.06.052. PubMed: 18662748. 18662748 10.1016/j.neuroscience.2008.06.052

[44] Hassani Z, O’Reilly J, Pearse Y, Stroemer P, Tang E et al. (2012) Human neural progenitor cell engraftment increases neurogenesis and microglial recruitment in the brain of rats with stroke. PLOS ONE 7: e50444. doi:10.1371/journal.pone.0050444. PubMed: 23185625. 23185625 10.1371/journal.pone.0050444 PMC3503964

[45] Trueman RC, Klein A, Lindgren HS, Lelos MJ, Dunnett SB (2013) Repair of the CNS using endogenous and transplanted neural stem cells. Curr Top Behav Neurosci 15: 357-398. PubMed: 22907556. 22907556 10.1007/7854_2012_223

[46] Wang X, Mao X, Xie L, Sun F, Greenberg DA et al. (2012) Conditional depletion of neurogenesis inhibits long-term recovery after experimental stroke in mice. PLOS ONE 7: e38932. doi:10.1371/journal.pone.0038932. PubMed: 22723908. 22723908 10.1371/journal.pone.0038932 PMC3378583

[47] Ducruet AF, Zacharia BE, Sosunov SA, Gigante PR, Yeh ML et al. (2012) Complement inhibition promotes endogenous neurogenesis and sustained anti-inflammatory neuroprotection following reperfused stroke. PLOS ONE 7: e38664. doi:10.1371/journal.pone.0038664. PubMed: 22761695. 22761695 10.1371/journal.pone.0038664 PMC3383680

[48] Zhao BQ, Wang S, Kim HY, Storrie H, Rosen BR et al. (2006) Role of matrix metalloproteinases in delayed cortical responses after stroke. Nat Med 12: 441-445. doi:10.1038/nm1387. PubMed: 16565723. 16565723 10.1038/nm1387

