# Supplementary figures and images for: Stem Cell Recruitment of Newly Formed Host Cells via a Successful Seduction? Filling the Gap between Neurogenic Niche and Injured Brain Site
Source: PLoS One. 2013 Sep 4;8(9):e74857. doi: 10.1371/journal.pone.0074857 (PMC3762783; doi:10.1371/journal.pone.0074857)

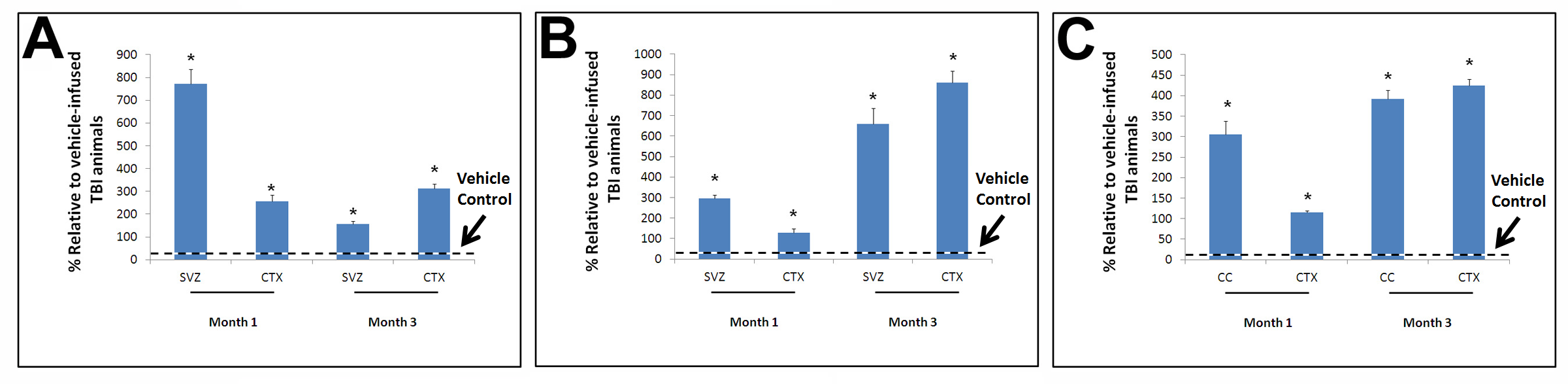

Supplement: Figure S1 — Quantifications of Ki67, nestin and DCX labeled cells are shown in panels A, B, and C, respectively. Asterisks (*) indicate significant increase in the number of phenotypically labeled cells counted per high-power field view (28,800 µm2) selected at random in the region of interest in TBI animals transplanted with SB623 cells compared to TBI animals that received vehicle only (p’s < 0.05). (TIF) [file pone.0074857.s001.tif]
